# Alcohol a Factor in Crime

**Published:** 1896-02

**Authors:** I. M. Quimby


### Abstracts and Selections.

### Alcohol a Factor in Crime.

It is but recently that the true nature and effects of alcohol, through the investigations of Drs. Richardson, Magnan, Norman Kerr, Almon, T. D. Crothers, J. H. Kellogg, N. S. Davis and others, have been fully demonstrated.

Alcohol should be no longer considered in any of its relations an innocent liquid, "a good creature of God or the milk of the aged, a stimulant, a true supporter of combustion, a promoter of life or health, a food or an aid to digestion, an element to sustain mental or physical powers, or a constructor of healthy cell growth or tissue," but on the contrary, it should be considered and known by all who persist in using it, as a narcotic from the beginning, as a multiplier and promoter of disease, in all their myriad forms, a destroyer of the blood and tissues, a destroyer of health, happiness and character, and through its paralyzing effect, a promoter and a great factor in crime.

It should be known and clearly stated that alcohol is not only a poison per se, and where taken into the system not only injures every organ and tissue, but through its operation has the power of producing a ptomaine or other micro-organism which is quite as destructive as alcohol itself. This ptomain results from the action of alcohol preventing proper excretion and elimination from the system of poisonous elements. Therefore the users of spirits carry in their system a dual poison, alcohol and a species of auto-intoxication, caused by alcohol, both or either of which are destructive to physical and mental powers.

The larger quantity of warm blood sent into the skin communicates a sensible glow, which feels to the person affected like an increased warmth of the body generally. The feeling of warmth and vigor is deceptive.

The feeling is as pleasant as it is delusive, as cheering as it is deceptive. It is not until we measure up the effects of this first stage, with the precison of an observer who is looking at the phenomena without feeling them, that the truth is made manifest. Then it comes out clearly that the over-action felt subjectively is waste without compensation, lost energy, and so lost that in no sense whatever is it regained. Alcohol has crippled, through its irritation, the normal mechanism of the body.

It narcotizes the mind as well as the body, hides from us the truth about ourselves, and makes us satisfied, when we ought to be ashamed.

The growth of inebriety and the alarming increase of this class of defectives, call loudly for public attention in their behalf. If the State licenses the saloon, the most destructive agent in modern society, why should she not also provide for the legitimate product of the saloon, the degenerate drunkards, and use license fees for the erection of inebriate insane asylums? And why should not laws be passed legalizing compulsory commitment to such institutions of those who have lost self-control and need the sheltering care and charity of the State, which not only has consented to, but has also been, a particeps criminis in their destruction?

Our present method of treating the victims of inebriety is barbarous in the extreme, if it be not positively criminal. The drunkard, weakened in every power, needs not so much consignment to a cell with common criminals, to be further demoralized, as to a hospital for healing, where the State, under proper medical advice, should legally incarcerate and hold him under treatment until he has been restored to normal health and power.

The regular appearance of the vast army of drunkards in our police courts, and the heartless and ignorant treatment they receive, is one of the foulest blots upon our boasted civilization. Let the State arise in her might for the legal protection and healing of her weak children, whose legal destruction she has authorized in the American saloons. The State has need to change her antiquated laws and methods, for those more in sympathy with the results of modern scientific investigation, and thus wisely use the right arm of her power in the cause of humanity, and for the liberation of the slaves whose chains she has helped to forge.

These numerous wine shops increase many forms of disease and multiply crime about fifty per cent. These facts are producing alarm among legislators, and the most stringent measures of repression are proposed; particularly to diminish the wine shops and confine the victims for treatment who persist in drinking.

But now since the action of alcohol on the human system is better understood in the light of modern science, how ridiculous appear all repressive or preventive laws against the vice of spirits on one hand, while on the other hand, laws are enacted permitting the exposure for sale of alcoholic compounds by the government!

It is unnecssary to pursue statistics to show that the alcohol not only destroys families, but is causing the nations to commit suicide. It is estimated that over fifty per cent of the births in France are illegitimates. According to statistics fifty to seventy-five per cent of all the crime committed in the United States is done by persons while under the influence of alcohol.

I turn from these great facts which are so clear to all observing persons, to the practical clinical side that comes into view in every community. How far does the anæsthetic effect of spirits appear in the next generation? I remarked to a gentleman once that alcohol should be termed the great breeder and crime promoter of the universe. He replied somewhat boastfully: "Why, doctor, I have drunk whiskey nearly every day for forty years; am now sixty-five years old, yet never was drunk in my life, or committed a wrong while under the influence of liquor, and have a good constitution yet."

This man told partly the truth, but to get at the whole truth you will have to study the history of his children, of whom he had four.

His oldest, a young man of twenty-five, of irregular habits and of questionable character.

The second, a young lady of twenty-two, of good health.

The third, a young lady of eighteen, somewhat brilliant, but epileptic, and in poor health.

The fourth, another young lady, of sixteen, with spinal curvature, and exceedingly nervous, irritable and troublesome, with general poor health.

Now, did the father's moderate, but continuous use of alcoholics and insidious palsy have anything to do with the sadly imperfect organization and development of his children?

The above facts may be duplicated many times, in almost any community. Run over the families of your acquaintances in which either father, mother, or both are moderate and constant drinkers, and observe for a number of years the health and actions of their children, and it will be seen that from fifty to seventy-five per cent will be defective.

From seventy-five to eighty per cent of crime is caused by the use of alcoholic liquors, and more than fifty per cent of poverty and wretchedness is caused by the use of sprits.

Death rates and diseases are doubled in every drinking community. In commnnities where liquors are most consumed, jails, almshouses and prisons multiply, ignorance prevails and crime increases.

Statistics have shown over and over again that the monomaniac, dipsomaniac, the idiot, the epileptic, the inebriate, and the feeble-minded, etc., are increasing at a greater ratio than the normal increase of the population.

The above facts being true, what excuse can there be to the healthy and unbiassed mind for the exhibit of alcoholic liquors in every hamlet, town and city of our country? For it is the week and feeble-minded and this class of defectives which largely compose the criminal class, who are mainly the patrons and supporters of the wine shops.

The conclusion which I would urge with emphasis is, alcohol is a paralyzant and a dangerously destructive agent, and the victim has not full possession of his faculties. He is not sound and normal. He is mentally crippled. He can not exercise the full powers of his brain, because it is palsied and under a mask. His perception and powers of reason are faulty. This can be measured and demonstrated by instruments of precision, and is no longer theory and opinion, but an absolute clinical fact.

From this we can date crime and all its associated evils with the same certainty that oaks will spring from acorns.

Also from this point of view the whole field of medical jurisprudence stretches out clearly, and all its confused theories are ranges of tenable facts that can be seen and prevented on the same plane as the germs of typhoid and typhus are removed, or treated.—*I. M. Quimby, M. D., in Medico-Legal Journal.*

